# Parenting Sense of Competence in Parents of Children With and Without Intellectual Disability

**DOI:** 10.5964/ejop.3771

**Published:** 2021-05-31

**Authors:** Sanja Jandrić, Ana Kurtović

**Affiliations:** a Unit for Child and Adolescent Psychiatry, Clinical Hospital Center, Osijek, Croatia; b Faculty of Medicine, J. J. Strossmayer University of Osijek, Osijek, Croatia; c Department of Psychology, Faculty of Humanties and Social Sciences, Osijek, Croatia; The Maria Grzegorzewska University, Warsaw, Poland

**Keywords:** parenting sense of competence, perceived stress, intellectual disability

## Abstract

Our study aims to examine the relationship of child’s intellectual disability, parental education, employment and perceived stress with parenting sense of competence (satisfaction and self-efficacy). Three groups of parents (children without intellectual disability, children with mild intellectual disabilities, and children with moderate/severe intellectual disability) completed measures of perceived stress, parenting sense of competence and socio-demographic questions. Results show that child’s intellectual disability affects parenting satisfaction but not parenting self-efficacy. Parental employment predicted parenting satisfaction, but not parenting self-efficacy, while perceived stress predicted parenting satisfaction and self-efficacy. Results further suggest that parental employment moderates the relationship of child's disability with parenting satisfaction and perceived stress. Result suggest a need for interventions aimed at supporting parents in dealing with emotional consequences of their child’s disability.

Children’s well-being is closely related to parents’ well-being, and most studies demonstrate that the quality of parental care, which greatly determines the child’s adjustment, is influenced by parents’ sense of competence in their role as parents (Coleman & Karraker, 1997; Elek, Hudson & Bouffard, 2003; Greenspan & Wieder 2003; Hudson, Elek, & Fleck, 2001; Johnston & Mash, 1989; Landry, Smith, & Swank, 2003; Priel & Besser, 2000; Sanders & Woolley, 2005). Parenting sense of competence is a construct derived from social—cognitive perspective (Bandura, 1982; Bandura & Cervone, 1983), which emphasizes cognitive processes in the development of personality. However, in research and measurement of parenting cognitions about their parenting, there has been quite a lot of differences and overlap between constructs of parenting competence, self-efficacy, and parenting self-esteem. Authors sometimes use these terms interchangeably and, even when they do not, the definitions of parenting self-efficacy and competence are very similar (Vance & Brandon, 2017). One of the reasons for this lack of conceptual clarity might stem from using different measurements and their theoretical backgrounds. While some studies focus on self-efficacy as one’s perception of one’s competency at performing different tasks, either in a task-specific (relating to specific tasks of parenting), domain-specific (relating to different tasks in a broader domain of parenting) or domain-general (a global competency expectation as a parent) (Boruszak-Kiziukiewicz & Kmita, 2020; Coleman & Hildebrandt Karraker, 2000; Wittkowski, Garrett, Calam, & Weisberg, 2017), others take a broader approach to conceptualizing parenting competence (Miklósi, Szabó, & Simon, 2017; Ohan, Leung, & Johnston, 2000; Rybski & Israel, 2017; Teti & Gelfand, 1991). This approach defines parental sense of competence in terms of parental self-esteem in accordance with multidimensional models of self-esteem (Harter, 1985; Tafarodi & Swann, 1995). This approach defines parenting competence as having two correlated but independent components—one referring to a sense of personal efficacy and one referring to a sense of one’s value and satisfaction as a parent. Therefore, parents, who feel competent in their parenting role, consider themselves in control of their parenting behavior, efficient in their ability as a parent, and they are satisfied with themselves as a parent (Grusec, Hastings, & Mammone, 1994; Miklósi et al., 2017; Ohan et al., 2000; Rybski & Israel, 2017; Sanders & Wooley, 2005; Sevigny & Loutzenhiser, 2010).

Parenting sense of competence is an important focus of both research and interventions aimed at preventing or alleviating problems behaviors in children (Holden, 2010; Kapetanovic, Skoog, Bohlin, & Gerdner, 2019; Latham, Mark, & Oliver, 2018). This seems to be especially important if a child is at risk or has special needs, due to greater demands and pressures that parents are faced with (Baxter, Cummins, & Yiolitis, 2000; Challela, 1981). Developmental disability in the family often affects the entire family system, usually because the mother has to provide extra care and time for the disabled child, leaving fewer resources for other responsibilities and relationships, within or outside the family (Lundy, 2011; Vanderkerken, Heyvaert, Onghena, & Maes, 2019). Children with intellectual disability process information more slowly than children without intellectual disability. They have difficulties communicating, managing life skills, and understanding abstract concepts (Minnes, 1998; Shaffer et al., 1985). According to the fifth edition of Diagnostic and statistical manual of mental disorders (DSM-5; American Psychiatric Association [APA], 2013) diagnosis of intellectual disability is warranted when a child has a deficit in intellectual functioning (e.g., reasoning, problem solving, abstract thinking etc.), and significant impairment in adaptive functioning. As opposed to older classifications, namely International Statistical Classification of Diseases and Related Health Problems (ICD-10; World Health Organization, 2009) and DSM-IV (APA, 1994), the severity of the disability is determined on the basis of deficits in adaptive functioning in three domains (conceptual, social and practical), and not on the basis of intellectual impairment or IQ score. Children with mild intellectual disability have difficulties in acquiring academic skills (such as reading, writing, calculus) and abstract thinking as they get older (APA, 2013, 2019). Their level of social and practical functioning is below what is expected for their age group. They are usually able to develop satisfactory relationships and basic self-care, but they require support in certain areas (such as social reasoning or self-protection) (Alexander & Reynolds, 2020; Platt, Keyes, McLaughlin, & Kaufman, 2019). Children with moderate intellectual disability display significant delay in acquisition of academic skills and require constant support (APA, 2013, 2019; Sermier Dessemontet, de Chambrier, Martinet, Moser, & Bayer, 2017). Similarly, their social functioning is characterized with significant deficits reading social cues and understanding relationships (APA, 2013, 2019; Platt et al., 2019), while in practical domain usually their competence is limited to basic self-care and requires a prolonged learning period (APA, 2013, 2019). Children with severe intellectual disability have very limited acquisition of academic skills, and limited understanding of written word. Their social functioning is severely limited in terms of vocabulary and syntax, and they require support and supervision in practically all everyday activities (APA, 2013, 2019).

Raising a child with intellectual disability brings a set of challenges for parents in terms of managing a growing need for care, availability of support and dealing with the child’s limitations (Challela, 1981; Hastings & Brown, 2002; Krauss, 1993; Salonen et al., 2009). Studies show that parents often feel fear, frustration, helplessness and guilt for not being able to help their child more (Baxter et al., 2000; Challela, 1981; Moos & Holahan, 2003), all of which can result in them feeling incompetent as parents and consequently affect parenting practices and child’s well-being. Parents who feel competent in their parenting tasks, on the other hand, are less likely to perceive their child as problematic, and more likely to be satisfied (Coleman & Hildebrant Karraker, 2000). Even though parents of children with intellectual disability report being satisfied with their children and that having a child with intellectual disability has had a positive impact on their self-efficacy and satisfaction (Bunga, Manchala, Tondehal, & Shankar, 2020; Gilmore & Cuskelly, 2012; MacInnes & Fraser, 2009), as well as personal growth, maturity and family life in general (Hastings, Allen, McDermott, & Still, 2002), there are also studies demonstrating differences in comparison to parents of children without disability. For example, Al-Kandari and Al-Qashan (2010) found that mothers of children with intellectual disability had exhibited lower levels of self-efficacy and had negative beliefs about their parental abilities, while Gilmore and Cuskelly (2012) found that parents adapt to their child’s needs over time, and their sense of competence, while lower at first, does increase as the child develops. Moreover, the effects on parental self-esteem do not necessarily come from the child’s disability per se, but rather from associated behavioral and emotional problems (Ohan et al., 2000).

Therefore, the link between characteristics of the child with intellectual disability and parenting sense of competence is not yet clear, given that studies show different results. This suggest that there are other factors, which might determine parental adjustment to their child’s disability and their sense of competence as a parent, apart from the disability itself. Indeed, studies suggest that there are socio-economic factors which might have a significant role (DeLongis & Holtzman, 2005; Jones & Prinz, 2005). Flynt and Wood (1989) demonstrated the effects of age, marital and socio-economic status on coping and adjustment of mothers of children with moderate intellectual disability. Employment status is related to better socio-economic status and thus has positive effects in terms of stress reduction (Cidav, Marcus, & Mandell, 2012; Saltzstein et al., 2001). However, in parents of children with intellectual disability, one parent is often unemployed due to increased need for care (either by choice or due to getting fired which is often the case in Croatia), which can put an extra strain on families’ resources, increase stress and affect adjustment. Studies in both Eastern and Western countries have shown that mothers of children with disabilities are unemployed more often than mothers of children without disabilities (Brown & Clark, 2017; Ejiri & Matsuzawa, 2019; Olsson & Hwang, 2006). Although a variety of factors affect employment status of parents of children with or without disability (Brown & Clark, 2017), data consistently shows that child’s disability is one of the factors associated with it. Also, Ouyang et al. (2014) found that parent of children with fragile X syndrome and parents of children with autism spectrum disorders and intellectual disability have more often quit their job or worked less in order to take care of the child, than parents of children with either autism spectrum disorder or intellectual disability. Similar differences were found by Saunders et al. (2015). These results suggest that a more severe disability is related to more unemployment and financial burden.

Education, on the other hand, was shown to be an important factor in parental adjustment to their child’s disability, mostly due to more knowledge, better understanding of the disability and more proactive attitudes and self-advocacy (Landry, Smith, & Swank, 2006; Miller, Gordon, Daniele, & Diller, 1992). Number of children in the family could affect parenting sense of competence in different ways. On one hand, previous experience in raising children has shown positive effects on parenting and family adjustment (Glidden, Flaherty, & McGlone, 2000). On the other hand, having more children, especially young ones close in age, can put an extra strain on parents, both financially and emotionally, as some studies suggest (Kwai-sang Yau & Li-Tsang, 1999; Skreden et al., 2012).

Finally, perceived stress was shown to be a major factor in explaining parenting sense of competence, parental behavior as well as overall adjustment in both parents of children with disabilities and in parents of typical children (Johnston & Mash, 1989; Panneton & Deater Deckard, 2017). The relationship between stress and parenthood has long since been a subject of research in developmental psychology (Crnic, Greenberg, Ragozin, Robinson, & Basham, 1983; Deater Deckard & Scarr, 1996; Hastings & Brown, 2002; Salonen et al., 2009). Research has mostly, focused on general sources of parenting stress, such as various social and economic factors (low income or unemployment), or child’s intellectual disability (Conger & Elder, 1994; Crnic et al., 1983; Demerouti et al., 2005; Whippl & Webster-Stratton, 1991), which, when accumulated, can decrease parents’ sense of efficacy and affect the quality of parent-child interactions (Crnic & Low, 2002; Deater Deckard, 1998; Gutermuth et al., 2005; Reece & Harkless, 1998). Indeed, studies show that daily stress is related to parenting behavior (Deater Deckard, 2005) and parenting competence (Deater Deckard, 1998). Studies also consistently show elevated levels of stress in parents of children with intellectual disability (Arakkathara & Bance, 2019; MacInnes & Fraser, 2009; Masulani-Mwale, Kauye, Gladstone, & Mathanga, 2018; Phillips, Conners, & Curtner-Smith, 2017; Tsermentseli & Kouklari, 2019), both mothers (Leung, 2019) and fathers (Marsh, Brown, & Mccann, 2020), although those levels are related solely to the child’s disability but also other factors, both internal (such as parents’ coping) end external (such as social support), as well as the child’s internalizing and externalizing problems (Barak-Levy & Atzaba-Poria, 2020; Hassall, Rose, & McDonald, 2005; Leung, 2019). Higher levels of stress in parents of children with intellectual disability put a significant strain on parents, increase the risk of mental health problems, marital problems and can impair parents’ ability to positively affect the child’s behavior, as well as decrease wellbeing and overall quality of family life (Arakkathara & Bance, 2019; Azeem et al., 2013; Barak-Levy & Atzaba-Poria, 2020; Langley, Totsika, & Hastings, 2020).

The focus of our study was to examine the effects of child’s intellectual disability, socio-demographic variables, and perceived stress on parenting sense of competence (satisfaction and self-efficacy) in a sample of parents in Croatia.

## Method

### Participants and Procedure

Participants were 107 parents of primary school children (79 mothers and 28 fathers), aged 28 to 57 years old (*M* = 41.86, *SD* = 5.652). The unequal number of mothers and fathers is not unexpected given the fact that involvement in child’s education as well as other childcare activities has been in favor of mothers, especially more traditional societies (Craig & Mullan, 2011). Croatia is still quite traditional in its understanding of gender roles, with mothers assuming more responsibility in childcare (Bartolac & Kamenov, 2013).

The majority of participants were married or in a civil union (89.7%), while 10.3% were divorced or widowed. The majority of parent had two or three children (77.5%), 13.1% had only one child, and 9.3% had more than three children. More than half of the participants (54.7%) have a high-school degree, 8.4% had primary school education, while 36.8% of the participants had a higher education degree (university or postgraduate studies). With regard to the employment, 67.9% of the participants were employed and 32.1% of them were unemployed or retired (there were no parents with part time employment).

There were three groups of parents; parents of children without intellectual disability (*N =* 35, 32.7%), parents of children with mild intellectual disability (*N =* 33, 30.85%), and parents of children with moderate/severe intellectual disability (*N* = 39, 36.4%). In all three groups, children were 7 to 14 years of age, with equally distributed genders, 15 to 17 girls, and 16 to 22 boys respectively. The diagnosis of intellectual disability was made independent of the study by an interdisciplinary team comprised of a psychiatrist a psychologist, an educational rehabilitator, a speech therapist, a pedagogist, a teacher and a school medicine specialists.

Inclusion criteria for mild intellectual disability group were: having children with diagnosed mild intellectual disability or borderline intellectual disability with comorbid specific developmental disorders (including but not limited to communication disorders, learning disorders, attention deficit hyperactivity disorder, or autism spectrum disorder—severity level 1) whose level of adaptive functioning is congruent with mild intellectual disability and require support in different domains of functioning. Inclusion criteria for moderate/severe group were: having children with diagnosed moderate or severe intellectual disability with or without comorbid severe developmental disorders (including but not limited to autism spectrum disorder—severity level 2 and 3, Rett syndrome, cerebral palsy). There were only five parents of children with diagnosed severe intellectual disability, and for that reason, the decision was made to merge parents of children with diagnosed mild and severe intellectual disability. Inclusion criteria for parents of children without intellectual disability was having a child without developmental disabilities. Additional inclusion criteria for all three groups of parents was giving informed, written consent for participation in the study. Exclusion criteria was having a child with serious psychiatric comorbidity (e.g., psychosis) and not providing informed, written consent. Inclusion and exclusion were made based on children’ medical record documentation.

The study was approved by institutional Ethics committee, and conducted in one elementary school and one special education institution in Croatia. Subsamples of parents of children without intellectual disability and parents of children with mild intellectual disability were examined in the elementary school where their children were enrolled, while subsample of parents of children with moderate/severe intellectual disability was examined in the special education institution were their children were enrolled.

Parents completed measures of parenting sense of competence, perceived stress and socio-demographic questions during group parent—teacher meetings. Participation was voluntary, written consent was obtained and participant were informed of the anonymity and the confidentiality of obtained data, as well as that they can terminate their involvement in the study at any time.

### Measures

Socio-demographic data were collected on age, gender, number of children, marital status, educational level and parental employment.

#### Parenting Sense of Competence Scale

Parenting sense of competence was measured using Parenting Sense of Competence Scale (PSOC; Gibaud-Wallston & Wandersman, 1978), translation by Gustović-Ercegovac (1992). It is a 17 item self-report measure of parenting satisfaction (9 items, e.g., “My talents and interests are in other areas, not in being a parent”) and self-efficacy (8 items, e.g., “I honestly believe I have the skills necessary to be a good mother/father to my child”). Participant are expected to judge their agreement with items regarding their experience as a parent on a 6-point scale (ranging from 1—*strongly disagree* to 6—*strongly agree*). Higher scores indicate higher satisfaction and self-efficacy. The scale has good internal consistency with coefficients ranging from .77 to .80 (Ohan et al., 2000). Cronbach alpha coefficients in our study were .71 for parenting satisfaction and .70 for self-efficacy for the entire sample. Reliability coefficients for parents of children without intellectual disability were .77 for parenting satisfaction and .83 for self-efficacy. Reliability coefficients for parents of children with mild intellectual disability were .79 for parenting satisfaction and .85 for self-efficacy. Reliability coefficients for parents of children with moderate/severe intellectual disability were .82 for parenting satisfaction and .91 for self-efficacy.

#### Perceived Stress

Perceived stress was measured using the Perceived Stress Scale-10 (PSS; Cohen, Kamarck, & Mermelstein, 1983), translation by Hudek-Knežević, Kardum, and Lesić (1999). PSS-10 is a 10 item self-report scale measuring the level of experienced stress during the previous month. Participants are expected to answer how often they experienced each sensation on a 5—point scale (ranging from 0—*never* to 4—*very often*). Higher scores indicate higher levels of perceived stress. The scale has shown good reliability both in English and Croatian with reliability coefficients ranging from .78 (Cohen et al., 1983) to .88 (Hudek-Knežević et al., 1999). Cronbach alpha in our study was .88 for the entire sample, .84 for parents of children without intellectual disability, .87 for parents of children with mild intellectual disability, and .83 for parents of children with moderate/severe intellectual disability.

## Results

Table 1 shows descriptive data for parenting satisfaction, self-efficacy, and perceived stress for the entire sample, while Table 2 shows descriptive data for parenting satisfaction, self-efficacy, and perceived stress across three groups of parents.

**Table 1:** Descriptive Data for Parenting Satisfaction, Parenting Self-Efficacy and Perceived Stress

Variable	*M*	*SD*	Observed range	Possible range
Parenting satisfaction	40.39	9.008	9–54	9–54
Parenting self-efficacy	35.10	6.920	14–48	8–48
Perceived stress	17.09	5.997	3–31	0–40

**Table 2:** Descriptive Data for Parenting Competence and Perceived Stress Across Three Groups of Parents

Variable	Parents of children without intellectual impairment (*N* = 35)			Parents of children with mild intellectual impairment (*N* = 33)			Parents of children with severe intellectual impairment (*N* = 39)		
	*M*	*SD*	Min-Max	*M*	*SD*	Min-Max	*M*	*SD*	Min-Max
Parenting satisfaction	44.00	6.22	32–52	39.52	8.40	22–54	37.60	10.77	9–54
Parenting self-efficacy	35.60	6.09	22–47	34.72	7.07	23–48	34.97	7.67	14–48
Perceived stress	15.06	5.06	4–27	18.30	7.07	3–31	17.92	5.48	3–26

As can be seen in both Table 1, descriptive data point to parents exhibiting moderate to high sense of parenting satisfaction and self-efficacy, and moderate levels of perceived stress. However, standard deviations and observed range suggest that parents differ in their perception of parenting competence and stress almost across the entire theoretical range (especially for parenting competence).

Similarly to the results for the entire sample, parents exhibiting moderate to high sense of parenting satisfaction and self-efficacy, and moderate levels of perceived stress, but measures of dispersion suggest that parents differ in their perception of parenting competence and stress.

In order to examine the relationship between child’s intellectual disability socio-demographic variables, perceived stress and parenting satisfaction and self-efficacy correlation analysis was performed. Socio-demographic variables were coded as follows; Gender: 1—male, 2—female; Marital status: 1—marriage/civil union, 2—widowed/divorced (there were no single parents); Parents’ education level: 1—primary school, 2—high school, 3—university/postgraduate, and parental employment: 1—yes, 2—no (there were no parents with part-time employment nor retired parents). According to child’s intellectual disability, groups of parents were coded as follows: 1—parents of children without intellectual disability, 2—parents of children with mild intellectual disability, and 3—parents of children with moderate/severe intellectual disability. In accordance with requirements for correlations between variables, we used point-biserial coefficient to test correlation of gender, parental employment and marital status with criterion variables (parenting satisfaction and self-efficacy), Spearman’s coefficient to test correlation of parents’ age, education level and child’s disability with criterion variables, and Pearson’s coefficient to test the correlation between Perceived stress and criterion variables. The results are presented in Table 3.

**Table 3:** Correlation Coefficients Between Observed Variables

Variable	1	2	3	4	5	6	7	8	9
1. Parents’ gender	-								
2. Parents’ age	−.20^*^	-							
3. Number of children	−.11	.32^**^	-						
4. Marital status	.13	−.11	.02	-					
5. Parents’ education level	−.16	.11	−.35^**^	−.15	-				
6. Parental employment	.05	.04	−.08	.10	−.24^*^	-			
7. Child's disability	.06	−.14	.00	.05^*^	−.21^***^	−.28^**^	-		
8. Perceived stress	−.07	.07	.04	.16	−.00	.12	.19^*^	-	
9. Parenting self-efficacy	−.13	−.16	.13	−.02	−.24^*^	.13	−.12	−.29^**^	-
10. Parenting satisfaction	.06	−.14	.00	−.05	.21^*^	−.28^**^	−.37^**^	−.53^**^	.15

*p < .05. **p < .01. ***p < .001.

As shown in Table 2, parenting satisfaction is negatively correlated with perceived stress, *r*(107) = −.53, *p* < .01, and parental employment, *r*(107) = −.28, *p* < .01, and positively correlated with parents’ education level, *r*(107) = .21, *p* < .05. Therefore, parents who perceive more stress, who are employed, and parents with higher education level, are also more satisfied with their parenting role. Parenting satisfaction is also negatively correlated with child’s disability, *r*(107) = −.37, *p* < .01. Parenting self-efficacy correlated significantly (negatively) with perceived stress, *r*(107) = −.29, *p* < .01, and parents’ educational level, *r*(107) = −.24, *p* < .05, but was not significantly correlated with child’s disability. In other words, parents who perceive more stress and have a higher educational level, feel less efficient in their parenting role. Other socio-demographic variables did not correlate significantly with parenting satisfaction nor self-efficacy. It is important to point to the fact that parents’ gender did not have significant correlations with either variable except parents’ age, which suggest that the unequal number of mothers and fathers in the sample most likely would not affect the results.

In order to examine the effects of child’s disability, socio-demographic variables and perceived stress on parenting satisfaction and efficacy, hierarchical regression analyses were performed. Child’s disability was entered in the first step, parent’s educational level and parental employment in the second, and perceived stress in the third step. Results are presented in Table 4 and Table 5.

**Table 4:** Results of Hierarchical Regression Analyses for Parenting Satisfaction

Predictor	β	Summary
Step 1		*R*² = .08**, *F* = 10.25**
Child’s disability	−0.30**	
Step 2		*R*² = .11 (*p* = .05), ∆*R*² = .03 (*p* = .05), *F* = 5.08**
Child’s disability	−0.10	
Parents’ education level	0.12	
Parental employment	−0.21*	
Step 3		*R*² = .33***, ∆*R*² = .22***, *F* = 13.63***
Child’s disability	0.49***	
Parents’ education level	0.12	
Parental employment	−0.16	
Perceived stress	−0.49***	

*p < .05. **p < .01. ***p < .001.

**Table 5:** Results of Hierarchical Regression Analyses for Parenting Self-Efficacy

Predictor	β	Summary
Step 1		*R*² = .01, *F* = 0.02
Child’s disability	0.01	
Step 2		*R*² = .06*, ∆*R*² = 05*, *F* = 3.07*
Child’s disability	0.30*	
Parents’ education level	−0.31**	
Parental employment	−0.11	
Step 3		*R*² = .17**, ∆*R*² = .11**, *F* = 5.73***
Child’s disability	0.06	
Parents’ education level	−0.31	
Parental employment	−0.15	
Perceived stress	−0.34**	

*p < .05. **p < .01. ***p < .001.

As can be seen in Table 4, child’s disability predicted parenting satisfaction in a sense that higher disability severity predicted lower satisfaction. Parents’ education level did not have significant effects, but parental employment predicted higher satisfaction. However, once parents’ education level and parental employment were entered in the equation, child’s disability was no longer significant, suggesting that child’s disability predicts lower parenting satisfaction because it predicts unemployment, which is further supported by positive, significant correlation between child’s disability and parental employment (see Table 2). Finally, perceived stress, in the third step, predicted lower parenting satisfaction. However, once perceived stress was entered in the equation, the effect of child’s disability became significant again, higher and of an opposite direction, while the effect of unemployment was no longer significant. Given the fact that perceived stress and employment were not significantly related, this suggest that there might be an interaction between child’s disability and parental employment affecting perceived stress, as well as parenting satisfaction. The model explained 33% of total variance of parenting satisfaction.

As shown in Table 5, child’s disability was not a significant predictor. However, once parental employment and parents’ education level were entered the hierarchical regression analysis (HRA), child’s intellectual disability (positively) and parents’ education level (negatively) were both significant predictors, which was not in accordance with correlational analysis. Correlations between child’s disability, parents’ education level and parental employment most likely led to the inflation of variance in the second step (suppression effect). Perceived stress, in the final step, contributed significantly to parenting self-efficacy in a negative direction. The model explained 17% of total variance of parenting satisfaction.

Due to changes in beta coefficients depending on variables entered in individual step of the HRA, which suggested interactions between child’s disability and parental employment we performed moderation analyses using Hayes’ (2009) process macro for parenting satisfaction and self-efficacy, as well as perceived stress. The results of the analyses are shown in Table 6.

**Table 6:** Results of Hayes’ Process for Simple Moderation Analyses

Predictor	Coefficient	*t*	*p*	95% CI
Parenting satisfaction				
Child's disability	−5.6623	−2.5010	.0140	[−10.1550, −1.1695]
Parental employment	3.4467	1.7056	.0912	[−0.5635, 7.4570]
Interaction	4.9158	2.2646	.0264	[0.5482, 7.3589]
Parenting self-efficacy				
Child's disability	−0.9330	−0.5351	.5938	[−4.3942, 2.5282]
Parental employment	−2.2802	−1.4029	.1639	[−5.5066, 0.9461]
Interaction	0.4922	0.2474	.8051	[−3.4567, 4.4411]
Perceived stress				
Child's disability	3.0139	3.0326	.0031	[1.0417, 4.9861]
Parental employment	−0.4985	−0.4066	.6852	[−2.9310, 1.9340]
Interaction	−2.5101	−1.9999	.0252	[−5.0008, −0.0194]

**Figure 1:**
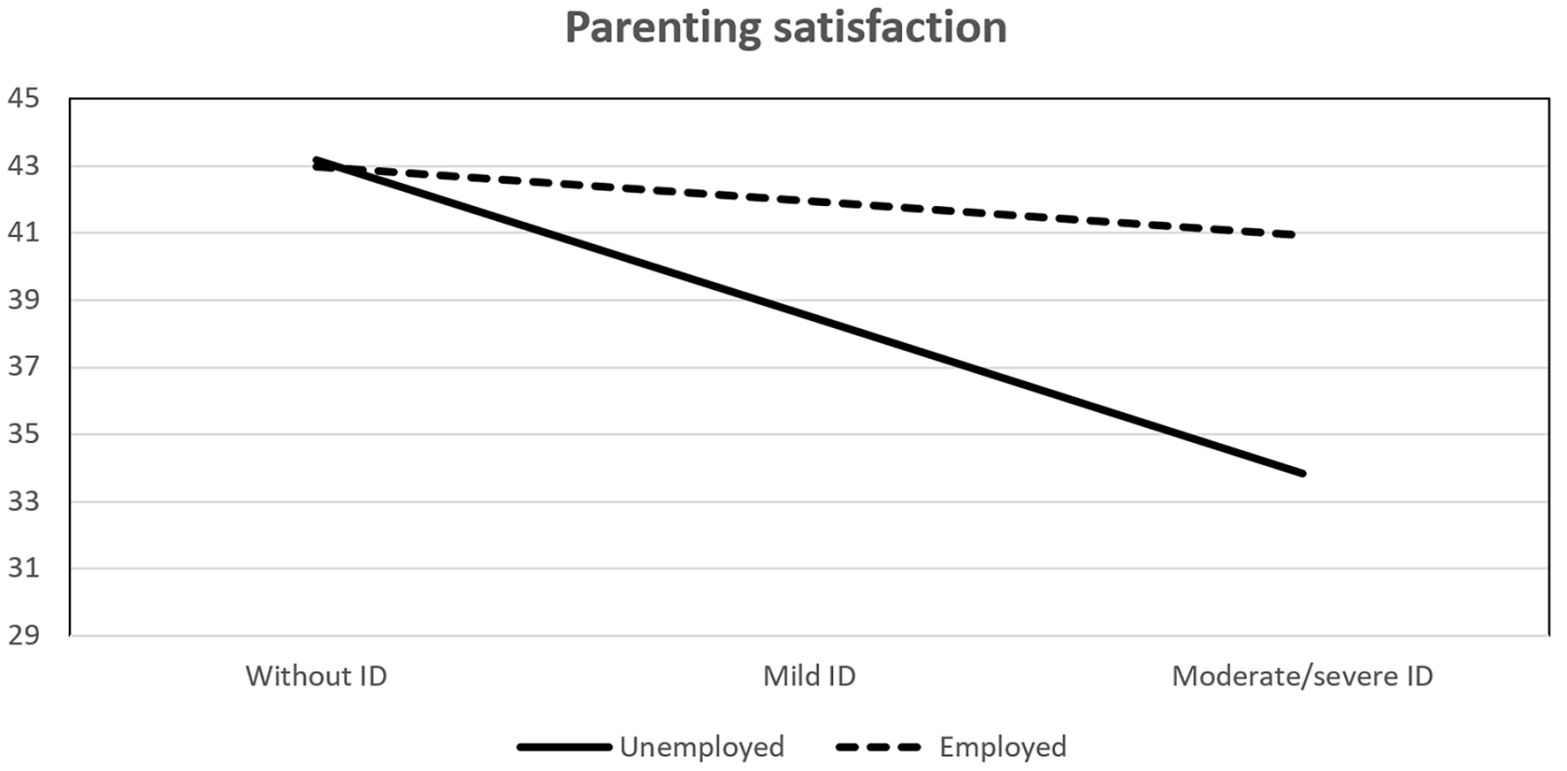
Effect of Interaction Between Child's Disability and Parental Employment on Parenting Satisfaction

**Figure 2:**
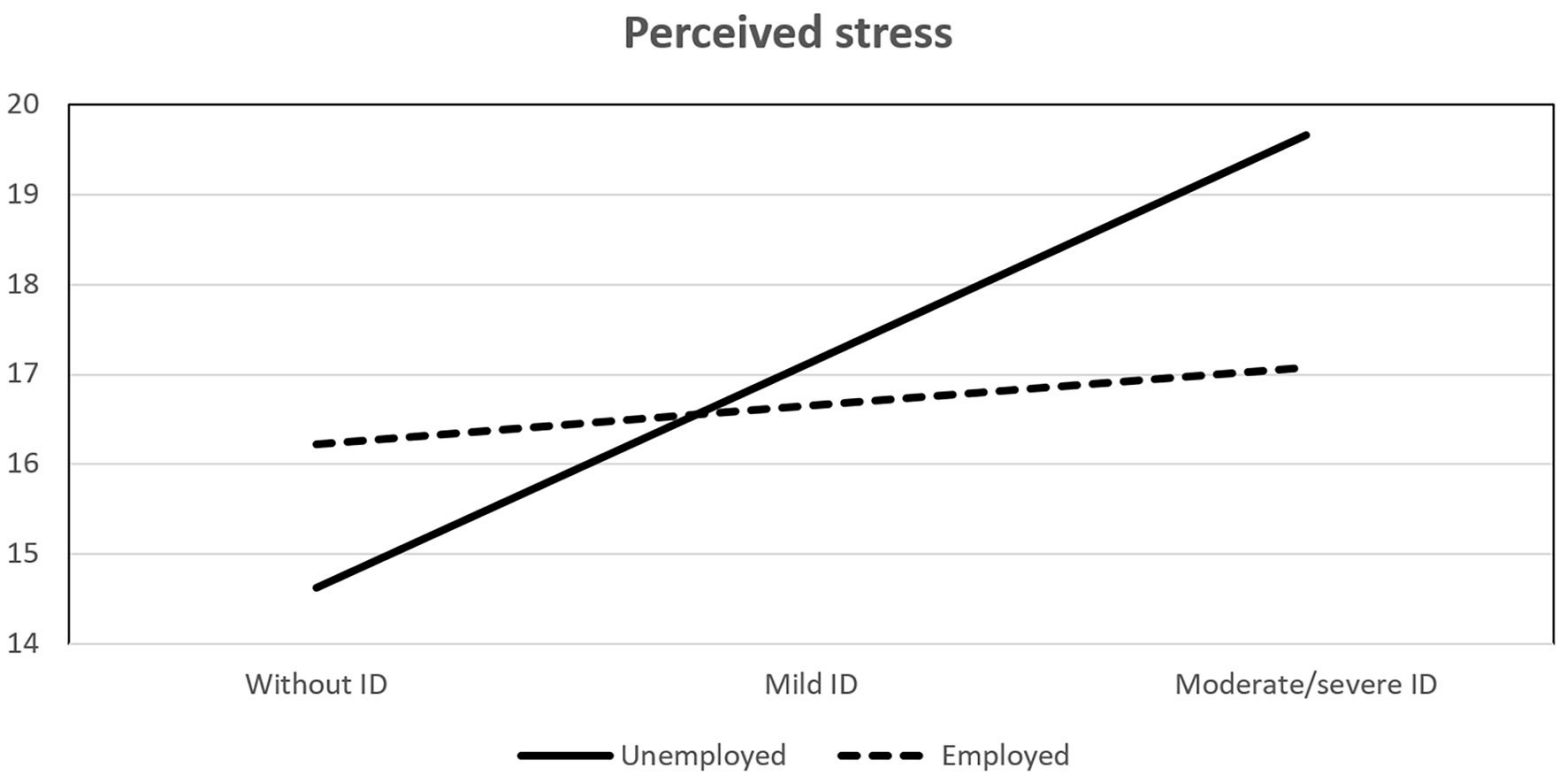
Effect of Interaction Between Child's Disability and Parental Employment on Perceived Stress

As can be seen in Table 6, the interaction effects of child’s disability and parental employment on parenting satisfaction and perceived stress were significant, while no significant effect was found regarding parenting self-efficacy. Analyses of conditional effects showed that the interactions were significant only in unemployed parents. In order to better understand the interactions, they are presented in Figures 1 and 2.

As can be seen in Figure 1, parents of children without intellectual disability do not differ in their satisfaction with respect to employment, while parents of children with intellectual disability do. The most pronounced difference is between employed and unemployed parents of children with moderate/severe intellectual disability. The unemployed parents are significantly less satisfied than the employed ones. Therefore, the differences are significant only in unemployed parents, suggesting a cumulative negative effect of unemployment and child’s disability on parental satisfaction.

As can be seen in Figure 2, unemployed parents of children with moderate/severe intellectual disability experience more stress that the employed ones, while the opposite is the case in parent of children with mild intellectual disability. The differences are also significant only in unemployed parents, suggesting that unemployment significantly increases stress in parents of children with moderate/severe intellectual disability.

## Discussion

Given the fact that, for most parents, satisfaction that they feel in their role as a parent greatly determines their satisfaction with life, the perception of competence as a parent is of vital importance for personal adjustment of the parents and their children (Arakkathara & Bance, 2019; Bunga et al., 2020; Langley et al., 2020), especially in parents of children with intellectual disability (Azeem et al., 2013; Lundy, 2011; Masulani-Mwale et al., 2018). With that in mind, our study has focused on examining the effects of the child’s intellectual disability on parenting sense of competence, as well as factors that might explain those effects.

Descriptive analysis has shown that, on average, parents feel satisfied and efficient in their parenting role. Although there were differences in parenting satisfaction between parents, as demonstrated by the range of the results, most parents feel confident about their knowledge, skills and abilities and find parenthood enjoyable and fulfilling. Descriptive data for perceived stress suggests that the majority of parents experience moderate levels of stress, although the range of results suggest that parent differ in their experience of stress.

Certain degree of concern about child behavior and development is an inevitable part of parenting (Hastings & Brown, 2002; Salonen et al., 2009). While those concerns may stem from parents’ sensitivity to their child's needs and motivate parents to meet those needs, they may also significantly increase stress if parents perceive that they cannot meet them (Deater Deckard, 2005).

Results of correlational analysis have shown correlations of child’s intellectual disability with parenting satisfaction, while it had no significant relations with parenting self-efficacy. Parents whose children have intellectual disability often encounter situations in which they feel helpless, frustrated and incompetent (Barak-Levy & Atzaba-Poria, 2020; Challela, 1981; Walden, Pistrang, & Joyce, 2000). In terms of developmental milestones, parents expect a child to be able to acquire new skills and competencies. Unfortunately, those expectations usually need to be changed and adapted, especially when it comes to children with intellectual disability whose parents often have trouble accepting their child’s limitations (Marsh et al., 2020). Continuous feelings of frustration and disappointment can lead parents to feel less satisfied with themselves as parents. Parenting self-efficacy, on the other hand, is more related to parents’ skills than child’s characteristics. Parents who feel efficient feel that they can solve problems related to their child and influence their behavior. It is less correlated with expectations from the child and more correlated with expectations from oneself, therefore understandably not correlated with the child’s disability (Jones & Prinz, 2005; Vance & Brandon, 2017).

Regarding socio-demographic factors, our results did not show significant relations between parents’ education level or parental employment, and perceived stress or parenting self-efficacy, but they did show that unemployed parents and less educated parents are less satisfied in their parenting role. This is in line with studies demonstrating positive effects of education on satisfaction with oneself, one’s life as well as parenting (Craig, 2006; Melin, Fugl-Meyer, & Fugl-Meyer, 2003; Orth, Trzesniewski, & Robins, 2010), and can also be indicative of economic pressures and parents feeling unable to sufficiently fulfil their child’s needs. Finally, parenting sense of self-efficacy has shown a negative correlation with perceived stress, which is consistent with earlier findings (Hanson, McLanahan, & Thomson, 1997; Masulani-Mwale et al., 2018; Tsermentseli & Kouklari, 2019) where parents with higher levels of stress estimated themselves to be less efficient parents.

Results of hierarchical regression analyses were in line with correlational analysis, for the most part. The only significant predictor of parenting self-efficacy was perceived stress, while, child’s disability, parental employment and perceives stress predicted satisfaction. The results are in line with previous studies demonstrating a negative relation of stress with parenting competence (Crnic & Low, 2002). Furthermore, they are in line with studies showing that long-lasting low-intensity stress (i. e. chronic daily stress) has a stronger effect on adjustment than major life stressors (Asselmann, Wittchen, Lieb, & Beesdo-Baum, 2017; Reece & Harkless, 1998), because Perceived stress scale used in our study is saturated more with day-to-day stress rather than with effects of major life events. Research has demonstrated that stress affects self-confidence, self-competence and self-esteem (Hudd et al., 2000; Johnston & Mash, 1989; Lazarus, 2000; Lazarus & Folkman, 1987, 2004; Reece & Harkless, 1998). Similarly, parents, who feel overwhelmed or not in control of their lives, are likely not to feel very satisfied with themselves as parent as well as in other areas of life (Abidin, 1992), and are likely to be less responsive to their child’s needs, which decreases their parenting sense of competence (Chavira, Wittchen, Lieb, & Beesdo-Baum, 2000; Miller et al., 1992; Rybski & Israel, 2017; Zukosky, 2009). Furthermore, our results suggest that stress has a weaker effect on parenting self-efficacy than it does on satisfaction. It is possible that even if a parent feels under stress, he or she still has to deal with obstacles and challenges of daily life, therefore may have the experience of successful coping and preserve a sense of self-efficacy. However, our result do suggest that the effects of stress on parenting competence are more emotional in nature, given the fact that parenting satisfaction reflects emotional experience of parenting, rather than a cognitive interpretation of ones skills and competence.

Finally, moderation analyses revealed effects of interaction between child’s intellectual disability and parental employment on parenting satisfaction and perceived stress. With regard to parenting satisfaction, parents of children without intellectual disability were the most satisfied with their parental role, regardless of their employment, while parents of children with moderate/severe intellectual disability who were unemployed were less satisfied than employed ones. Apart from the added economic burden of unemployment, which could explain the difference, it is also possible that unemployed parents are too immersed in their child’s life and lack balance in their own lives in terms of other sources of pleasure and satisfaction. Also, employed parents, due to better resources, could provide their child with extra help, as well as hire someone to help with everyday responsibilities, which can reduce stress and caregiver strain, as well as enable a parent to dedicate more quality time to their child, thus improving satisfaction. In support of that assumption, interaction of child’s intellectual disability and parental employment showed significant effect on perceived stress as well. Unemployed parents of children with moderate/severe intellectual disability experience significantly more stress than employed parents, while the opposite was true in parents of children without intellectual disability. Taken together, these results suggest a protective role of employment, at least in parents of children with moderate/severe intellectual disability. Similar results were found by Brown and Clark (2017), and Morris (2012) who’s results suggest that working mothers of school-aged children with disabilities have better work-family balance and mental health, as well as lower stress experience than unemployed mothers.

Our study offers some useful insights into the effects of child’s intellectual disability on parenting satisfaction and perceived stress in parents. First, they suggest that parents of children with intellectual disability could benefit from interventions aimed at promoting their sense of satisfaction in their parental role. Given the fact that satisfaction is a very subjective outcome, interventions should focus on providing support in dealing with emotional consequences of their child having an intellectual disability, accepting the reality of the disability while not overprotecting the child and trying to compensate for things that are not under their control. In dealing with emotional consequences, parents could also benefit from re-examining their expectations from themselves and others in order to have a more realistic view of whether they are actually providing their child with everything that is in their power to provide, as well as differentiating between what is under their control and what is not. Given the fact that our results suggest that greater perceived stress negatively affects parenting satisfaction and self-efficacy, interventions aimed at managing stress, such as coping skills training, relaxation techniques as well as support groups could, also, prove to be useful.

Improving availability of information about intellectual disability per se, as well as about different options and adaptations (through workshops, informal education etc.) could reduce stress and make parent feel more in control of what happens to their child. Unfortunately, in Croatia, in the region where the study was conducted, there are few services available to children with disabilities, especially in rural areas, and virtually none, which provide support to parents.

Furthermore, our results have implications for policy makers, primarily regarding employment. Our result show that unemployed parents of children with moderate/severe intellectual disability are under greater stress and less satisfied with themselves as parents. Measures that encourage employment of parents with children with developmental disabilities and discourage illegal, but nevertheless present, practice of discriminating against parents (especially mothers) who have children with disabilities should be taken, as well as promoting more flexibility in the work place which might improve work-family balance, even when their child requires more support.

Some limitations of our study should be mentioned. First, correlational and cross-sectional nature of the design prevents us from making causal conclusions as well as conclusions about whether the child’s intellectual disability, parental employment or perceived stress temporally precede changes in parenting competence. Parenting competence and perceived stress were measured using self-report measures, which can be confounded by socially desirable responses, especially when it comes to parental satisfaction, which is a sensitive subject for most parents and it is likely that parents would be less likely to report being dissatisfied in their parental role. Small sample size could also be an issue in limiting the possibility of generalization of result. Even though disproportion of mothers and fathers in the sample most likely did not affect the results, future studies should include more fathers in order to examine the role of parents’ gender.
